# Accumbofrontal tract integrity is related to early life adversity and feedback learning

**DOI:** 10.1038/s41386-021-01129-9

**Published:** 2021-09-24

**Authors:** Bryan V. Kennedy, Jamie L. Hanson, Nicholas J. Buser, Wouter van den Bos, Karen D. Rudolph, Richard J. Davidson, Seth D. Pollak

**Affiliations:** 1 University of Pittsburgh, Pittsburgh, PA USA; 2 University of Amsterdam, Amsterdam, Netherlands; 3 University of Illinois Urbana-Champaign, Champaign, IL USA; 4 University of Wisconsin-Madison, Madison, WI USA

**Keywords:** Human behaviour, Stress and resilience

## Abstract

Abuse, neglect, exposure to violence, and other forms of early life adversity (ELA) are incredibly common and significantly impact physical and mental development. While important progress has been made in understanding the impacts of ELA on behavior and the brain, the preponderance of past work has primarily centered on threat processing and vigilance while ignoring other potentially critical neurobehavioral processes, such as reward-responsiveness and learning. To advance our understanding of potential mechanisms linking ELA and poor mental health, we center in on structural connectivity of the corticostriatal circuit, specifically accumbofrontal white matter tracts. Here, in a sample of 77 youth (Mean age = 181 months), we leveraged rigorous measures of ELA, strong diffusion neuroimaging methodology, and computational modeling of reward learning. Linking these different forms of data, we hypothesized that higher ELA would be related to lower quantitative anisotropy in accumbofrontal white matter. Furthermore, we predicted that lower accumbofrontal quantitative anisotropy would be related to differences in reward learning. Our primary predictions were confirmed, but similar patterns were not seen in control white matter tracts outside of the corticostriatal circuit. Examined collectively, our work is one of the first projects to connect ELA to neural and behavioral alterations in reward-learning, a critical potential mechanism linking adversity to later developmental challenges. This could potentially provide windows of opportunity to address the effects of ELA through interventions and preventative programming.

## Introduction

Early life adversity (ELA) encompasses many different kinds of challenging experiences that a child might encounter, including abuse, neglect, exposure to violence, and limited family resources [1]. Nearly 40% of children endure multiple forms of adversity, and these experiences are associated with a host of negative outcomes including depression, anxiety, substance abuse, and educational underachievement [2, 3]. Neuroimaging research has been largely focused on the relationship between childhood adversity and threat processing. As expected, these studies suggest adversity is associated with structural and functional development of the hippocampus and amygdala [4–7]. While this focus on threat and vigilance processing is reasonable, less research activity has been directed at other behavioral challenges often associated with childhood adversity and the neurobiological mechanisms that might underlie these problems. One such area of concern involves the development of reward processing and learning deficits as a sequelae of ELA [8–13]. Alterations in reward processing and learning may relate to challenges commonly seen after adversity, including issues with learning and social functioning, but also potentially forms of psychopathology and poor mental health. Motivated by these ideas, we sought to address this gap in knowledge by examining corticostriatal neurobiology, which is critical to motivation, reward responsiveness, and learning.

The corticostriatal circuit includes the ventral striatum (VS), ventral tegmental area, and different portions of the medial prefrontal cortex (mPFC). This brain circuit is rich in the reward-related neurotransmitter dopamine and undergirds multiple aspects of reward reactivity and learning, such as the processing of primary, abstract, and perceived rewards [14–17]. Some reports suggest that childhood adversity is associated with volumetric reductions in the mPFC [18, 19], as well as structural and functional alterations in the VS [20–22]. But little is known about whether early adversity alters the white matter tracts connecting these areas. Newer MRI research techniques, such as diffusion-weighted imaging (DWI), allows a direct analysis of microstructural differences in white matter by mapping the three-dimensional diffusion of water through brain tissue [23, 24]. DWI metrics are sensitive to white matter differences like axonal density and ordering, myelination differences, as well as other properties [25]; together these factors are collectively referred to as white matter integrity.

The white matter pathway between mPFC and VS is termed the Accumbofrontal Tract. This tract’s white matter integrity is related to reward learning ability, as well as sensitivity to positive and negative feedback [26–28]. Greater white matter integrity, as index by fractional anisotropy, is related to better performance on reward learning tasks, as well as lower impulsivity and a higher willingness to delay reward [29–31]. Accumbofrontal tract connectivity is still developing through adolescence and into adulthood [32, 33]. ELA impacts the hypothalamic–pituitary-adrenal axis and stress-responsivity systems [34, 35], which may then alter reward-related, dopaminergic functioning, as well as white matter development in the developing brain [36, 37]. Therefore, a protracted period of postnatal neurodevelopment may leave the integrity of these tracts especially vulnerable to the effects of childhood adversity.

## The present study

Here, we examine the impact of earlier childhood adversity on white matter connectivity among adolescents. Given the behavioral problems in reward processing that have been associated with childhood adversity, we focused on the connection between the VS and the mPFC. To do so, we employed a broad and well-validated measure of childhood adversity, as well as state-of-science assays for quantifying white matter integrity, specifically quantitative anisotropy (QA). QA is a derived scalar metric that measures the anisotropy of water along a white matter fiber and has been shown to be a more accurate metric compared to other commonly used DWI metrics [38, 39]. Based upon observations of neural and behavioral decrements in reward circuitry among children with high levels of adversity (e.g., [9, 10]), our primary hypothesis was that higher levels of adversity would be related to lower white matter integrity in Accumbofrontal tracts. If confirmed, this would suggest that ELA influences critical corticostriatal neurobiology, specifically structural connectivity between the VS and mPFC. If this hypothesis was supported, we sought to test a second hypothesis regarding the behavioral relevance of these potential neurobiological differences. Specifically, we predicted that lower white matter integrity would be related to maladaptive decision-making processes as indexed by abnormalities in either positive or negative feedback reward sensitivity. Collectively, these hypotheses would connect experiences of ELA with neural and behavioral alterations in feedback sensitivity, a critical potential mechanism linking adversity to later developmental challenges.

## Methods

### Participants

Seventy-seven participants (39 female, 38 males) between the ages of 12 and 17 years (*M* age = 181 +/− 15.2 months; ~15 years of age) were recruited for this project. Participants were recruited from posting of flyers in the community. Parental consent and minor assent for adolescents was obtained for all participants and procedures were approved by the Institutional Review Board of the University of Wisconsin–Madison. The sample exhibited reasonable racial and ethnic diversity, with forty-seven participants (61%) self-identifying as non-Hispanic white, nineteen participants (25%) as Black/African American, eight participants (10%) as multiracial, two participants (2.6%) as Hispanic white, and one participant (1.3%) as Native American. Our sample also exhibited sufficient socioeconomic variations, as indexed by the Hollingshead Four-Factor Index [40]. The sample’s mean Hollingshead score was 42.97 (standard deviation = 15.7; range = 11–63.5). Of note, scores on this measure can range from 8 to 66 with higher values representing higher parental education and occupational prestige. On average, caregivers in our sample graduated from a 4-year college, and typically worked as social workers, teachers, nurses, or clerical and sales staff. Twenty-seven participants (35.1%) were from single-caregiver households. Table 1 detailed our sample’s demographics. Participants completed an MRI scanning session, an interview about life adversity, and a probabilistic reward-learning paradigm. A portion of the data from the behavioral paradigm from these participants was reported in [9].

**Table 1:** Demographic table with information and summary statistics about our sample, with distributions of age (in months), sex, and race reported.

Sample characteristics, *N* = 77*		
Age (Months)		*M* = 181 (SD = 15)
Sex	Male/Female	38/39
Race/Ethnicity		
	White, non-hispanic	47 (61%)
	Black/African American	19 (25%)
	Multiracial	8 (10%)
	White, Hispanic	2 (2.6%)
	Native American	1 (1.3%)
Early adversity	(from YLSI)	*M* = 3.78 (SD = 2.26)
	[Missing]	2
Hollingshead	(SES Composite)	*M* = 43 (SD = 16)
	[Missing]	3

Scores for our early adversity measure were derived from the YLSI and ranged from 1 to 9 in our cohort (with the maximum possible score of 10). The Hollingshead Four-Factor Index was used as a composite of socioeconomic status; this measure can range from 8 to 66 with higher values representing higher parental education and occupational prestige.

### MRI scanning session, acquisition parameters

Subjects completed an MRI scan on a 3.0 Tesla GE SIGNA (Discovery MR750) scanner with an 8-channel array head coil. DWI was performed using a diffusion-weighted, spin-echo, echo-planar imaging sequence with 48 non-collinear encoding directions at DW b = 1000 s mm^−2^. Eight additional non-DW (b = 0 s mm^−2^) images were acquired as reference volumes. Other protocol parameters were TR/TE = 8000/66.2 ms; parallel imaging (ASSET with acceleration = 2); flip angle = 90°; isotropic 2 mm resolution (128 × 128 matrix with 256 mm field-of-view). Seventy-four contiguous slices (2-mm thick) were prescribed axially, covering the entire brain. Anatomical (T1-weighted, 1 mm^3^) images were then acquired using a high-resolution 3-D, inversion recovery prepped fast spin-echo image with the following parameters: TE = 3.18 ms, TR = 8.13 ms, TI = 450 ms, flip angle = 12°, slice thickness = 1.0 mm.

### DWI preprocessing and tractography

**Fig. 1:**
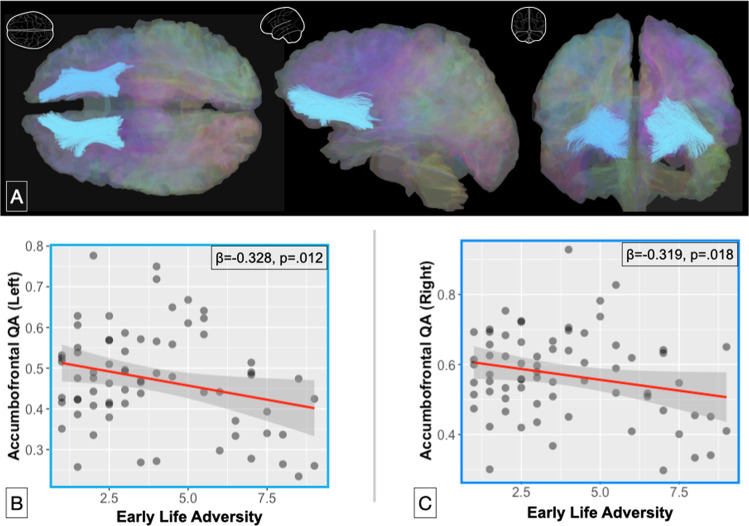
Visualization of our white matter tracts of interest. Our accumbofrontal tract of interest is shown in light blue (**A**; top), as well as associations between early life adversity and accumbofrontal quantitative anisotropy [QA] (**B**, **C**; bottom). Scatterplots for the left (**B**) and right (**C**) accumbofrontal tracts are depicted separately.

Diffusion-weighted images were then preprocessed for quality control and to maximize signal-to-noise ratio (e.g., mrtrix3 denoising; DSI-Studio’s B-table [41, 42]). DSI-Studio was then used for all DWI analysis, with reconstruction using the Q-space diffeomorphic method [43] and deterministic tractography. Two participants were excluded from further analysis due to poor neighboring voxel correlations. To probe variations in the Accombofrontal tracts, a tract-based mask was constructed from the Human Connectome Project’s population averaged (1 mm) template of 1065 subjects (HCP-1065; [44]). Specifically, we used: (1) left and right VS (from Freesurfer atlases) as seeds, (2) a region of interest (ROI) along the coronal slice one-third of the distance from the VS to the most polar region of the prefrontal cortex (PFC) to exclude tracts terminating prior to the PFC, and (3) regions of avoidance along the longitudinal fissure to ensure tracts remained within their respective hemispheres, and posterior to the VS, to ensure tracts traveled anteriorly. Tractography used streamlined orientation distribution function with the following settings: growth step size of 0.5 mm, max turning angle of 50°, 20% weighting smoothing at each step (from the previous step’s fiber direction), tract length between 20 and 85 mm to minimize anatomically implausible tracts, and termination when next fiber growth-step dropped below 0.25. ROI selection and other tractography settings were chosen based on consultation with local diffusion imaging experts and based upon prior research [26, 45]. These Accombofrontal tracts are shown in Fig. 1A. After both Accumbofrontal tracts were generated (one per hemisphere), they were used to extract values for individual participants.

DSI-Studio automatic fiber tracking was also used to generate control tracts in each hemisphere [44]; this was used to test if there were broad white matter alterations, outside of the corticostriatal circuit related to ELA and reward learning. The Middle Longitudinal Fasciculus was selected for the control tract because it is not directly involved in reward learning; instead, the Middle Longitudinal Fasciculus connects the superior temporal gyrus to the angular gyrus, playing a central role in language [46] and the integration of higher-order auditory and audiovisual functions [47]. Tractography settings were the same as for the accumbofrontal tract, except that the maximum length was changed to 200 mm and the max turning angle was set to 80° after consultation with the DSI-Studio software developers and expert users.

### Assessment of adversity

The lifetime adversity section of the Youth Life Stress Interview (YLSI) was administered separately to youth and their parents [48–50]. General and specific probes were used to assess a youth’s exposure to particularly stressful events and circumstances (e.g., death of a close family member or friend, exposure to severe marital conflict, and severe chronic illness of a close family member or friend). Semi-structured follow-up questions were then asked to assess the context surrounding each event. Interviews were scored by an independent team who generated a consensual rating on a 10-point scale. This coding incorporated consideration of the context of events and the impact on the child’s life rather than simply reflecting the sum of the number of stressors. As illustrative examples, a score of a 1 was given to a youth whose pet was hit by a car but was not seriously injured, a score of a 5 was given to a youth who was in foster care early in life, had multiple moves, and also had one of their parents die early in life, and a score of a 10 was given to a youth who was homeless, had several close family members die unexpectedly, and whose parents had a highly conflicted relationship that resulted in separation. A key point is that the scores not only reflect the objective stressors but also the subjective impact of these events as perceived by the youth. This rating system has high reliability and validity [49].

### Reinforcement learning behavioral paradigm

Participants completed a probabilistic reinforcement learning (RL) task while completing their scanning session. For this paradigm, participants saw two color drawings of everyday objects (e.g., a bell; a bottle) and were instructed to choose one by pressing a button corresponding to the stimulus on the left or right side [51]. Stimuli were presented for a maximum of 2500 ms. and offset after participant response. After their choice, participants received positive or negative feedback for 1000 ms. Feedback was delivered with two different, randomized probabilistic schedules, either AB or CD pairs. In AB pairs, the choice of stimulus A led to positive feedback on 80% of trials and stimulus B led to positive feedback on 20% of trials. In CD pairs, stimulus C led to positive feedback on 70% of trials and stimulus D led to positive feedback on 30% of trials. Feedback was given on every trial, except if no response was given within 2500 ms.; in these cases, the text ‘Too Slow’ was presented on the screen after stimulus offset. Participants were instructed to earn as many points as possible but were also informed that it was not possible to receive positive feedback on every trial. Receiving a positive feedback signal indicated earning of points. Beforehand, each participant completed 50 practice rounds to ensure that they understood the task. Participants completed two runs of 100 trials (50 AB pairs; 50 CD pairs). Each run consisted of different sets of pictures during which participants learned to choose stimuli A and C more often than stimuli B and D. The stimuli were presented in pseudorandom order with a jittered interstimulus interval (minimum = 1000 ms, maximum = 6000 ms). Stimuli were presented using E-Prime software (Psychology Software Tools, Pittsburgh, PA) with a screen resolution of 800 × 600 pixels.

### General cognitive ability

To help ensure that effects were specific to reward-learning rather than reflective of general cognitive processes, participants completed the spatial working memory task from the Cambridge Neuropsychological Test Automated Battery (CANTAB; Cambridge Cognition; Cambridge, UK). The CANTAB is computerized for standardized administration and does not require verbal responses. A spatial working memory score was calculated for each participant for the total number of errors during the task and z-transformed based on norms for each subject’s age and sex. This was used as a proxy of general cognitive ability.

### Youth behavioral problems

To characterize problem behaviors, caregivers completed the Child Behavior Checklist (CBCL; [52]), a widely used measure to assess child behavioral and emotional problems (e.g., [53, 54]). This 113-item scale asks about issues with anxiety, depression, social withdrawal, conflict with others and violation of social norms on a three-point Likert scale (0 = Absent, 1 = Occurs sometimes, 2 = Occurs often). Responses are normed for the youth’s age and gender and can be used to identify youth with scores in the elevated/clinically relevant range (>95 percentile) for internalizing and externalizing problems.

### Mathematical modeling of learning

To assess subcomponents of reward learning, a RL model was fit to each participant’s behavioral data [55]. This approach is commonly employed in decision-making research with adults [56, 57]. RL models use the prediction error (δ) to update the decision weights (w) associated with each stimulus (in this case A, B, C, or D). Thus, whenever feedback is better than expected, the model will generate a positive prediction error, which is used to “increase” the decision weight of the chosen stimulus (e.g., stimulus A). However, when feedback is worse than expected, the model will generate a negative prediction error, which is used to “decrease” the decision weight of the chosen stimulus (e.g., stimulus B). The impact of the prediction error is scaled by a feedback sensitivity parameter (α), which we calculated for positive feedback (α pos) and negative feedback (α neg). Additional information about our RL modeling is noted in our supplemental materials.

### Statistical analyses

Regression models were constructed to examine how stress exposure related to white matter integrity for both the accombofrontal tract (left and right entered separately in two different models) and a control tract (the middle longitudinal fasciculus). We entered adversity scores from the YLSI interview as our independent variable. We then completed two inter-related sets of analyses—first, we were interested if there were associations between tract integrity and feedback sensitivity (α pos or neg); and then if tract integrity played a mediating role in connections between stress and feedback sensitivity. These multiple statistical tests were adjusted using the Benjamini and Hochberg false discovery rate correction [58]. Related to mediation, we planned to probe potential mediation even if direct paths (between stress and feedback sensitivity) were non-significant given that important indirect effects can exist in the absence of direct effects [59–61]. This statistical testing of mediation was done using nonparametric bootstrapping in R, with 95% confidence intervals for indirect (a × b; a: stress-white matter, b: white matter-feedback sensitivity) effects. All models were adjusted for age (in months), race (binary coded as whether a participant was a Person of color, or not), general cognitive ability, and sex. Finally, post hoc exploratory analyses involving non-linear models of “stress inoculation” [62] were completed and are detailed in the Supplemental Materials.

## Results

### Descriptive statistics of the sample

As noted in Table 1, our sample experienced a modest amount of early adversity, as assessed by the lifetime adversity section of the YLSI. The mean adversity score was 3.78 (SD = 2.26) with a range from 1 to 9 (out of 10). Contextualizing this average in our sample, it was common for youth receiving scores of a 3 to have experienced serious marital conflict in their households or potential parental separation, as well as parental unemployment and challenges associated with that life event. Approximately 18% of our sample have scores of 6 or greater and this is similar to past reports from our group [50]. Youth receiving scores of 6 often have experienced parental mental health issues (i.e., alcoholism; chronic depression), caregivers divorcing, family or close friends passing away, and witnessing violence inside or outside of the home. Related to youth behavioral problems, 19.7% of participants indicated clinically relevant internalizing problems and 16.9% indicated clinically relevant externalizing problems on the CBCL.

### White matter tract integrity and childhood adversity

To examine the impact of adversity on corticostriatal white matter tract integrity, we examined associations between YLSI scores and QA metrics for the left and right accumbofrontal tracts. Childhood adversity was related to accumbofrontal tract integrity in both the left (*β* = −0.328, *p* = 0.012, *p*_*fdr*_ = 0.032) and right (*β* = −0.319, *p* = 0.018, *p*_*fdr*_ = 0.036) hemispheres. As predicted, greater adversity was associated with lower tract integrity. These associations are shown in Fig. 1. These relations remained significant (all *p*’s < 0.050) when controlling for general cognitive ability. To ascertain specificity in this finding, we examined the middle longitudinal fasciculus, which is outside of the corticostriatal circuit. Higher adversity was related to lower tract integrity in the right (*β* = −0.253, *p* = 0.0047, *p*_*fdr*_=  0.075) but not the left hemisphere (*β* = −0.169, *p* = 0.212) for this tract; however, no significant relationships were maintained when controlling for cognitive functioning (all *p*’s > 0.330).

### White matter tract integrity and feedback sensitivity

**Fig. 2:**
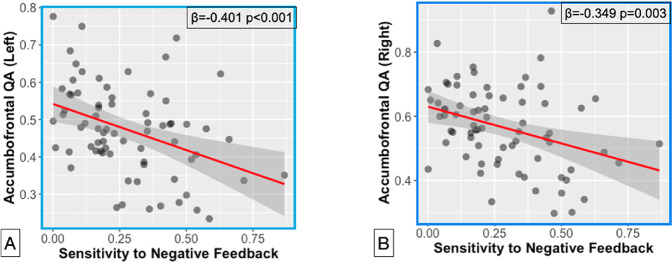
Associations between white matter and feedback learning. Scatterplots here show accumbofrontal quantitative anisotropy (vertical axis) and sensitivity to negative feedback (horizontal axis) for the accumbofrontal tract in the left (**A**) and right (**B**) hemispheres.

We next sought to examine if white matter integrity was related to sensitivity to positive and negative feedback during reward learning. To do so, we constructed separate regression models for each valence of feedback. QA metrics indicated that lower white matter integrity for the left and right accumbofrontal tracts were both related to greater sensitivity to negative feedback (Left accumbofrontal tract, *β* = −0.401 *p* = 0.0008, *p*_*fdr*_ = 0.0064; right accumbofrontal tract, *β* = −0.349 *p* = 0.0032, *p*_*fdr*_ = 0.0128). These associations are shown in Fig. 2. These results were maintained when controlling for cognitive ability (Left accumbofrontal tract, *p* = 0.0017; right accumbofrontal tract, *p* = 0.01). This suggests that aspects of learning, specifically negative feedback sensitivity, as opposed to attentional or other processes, are related to accumbofrontal white matter integrity. There were no associations between accumbofrontal tract integrity and positive feedback (all *p*’s > 0.262). Examining our control tract, Middle Longitudinal Fasciculus, did not reveal any associations between tract integrity and sensitivity to positive (all *p*’s > 0.7) or negative (all *p*’s > 0.64) feedback.

### Prediction of feedback sensitivity through white matter and early life adversity

**Fig. 3:**
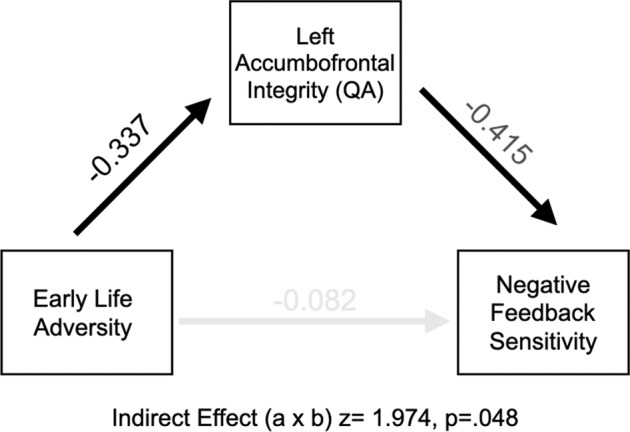
A path diagram depicting relations between early life adversity, corticostriatal neurobiology, and reward-learning. We used nonparametric bootstrap mediation models to test connections between ELA (X), feedback sensitivity (Y), and accumbofrontal tract integrity (M). These models indicated a significant indirect effect (a × b) of early life adversity related to accumbofrontal integrity and accumbofrontal integrity relating to sensitivity to negative feedback (*B* = 0.01, SE = 0.006, *z* = 1.974, *p* = 0.048; 95% CI = 0.002–0.025).

Given connections between ELA, white matter, and reward learning, we tested for potential statistical mediation by entering childhood adversity (X), feedback sensitivity on the reward learning task (Y), and accumbofrontal tract integrity (M) into nonparametric bootstrapped models in R’s ‘*lavaan’*. We did this separately for the left and right Accumbofrontal tracts. The direct association between adversity and sensitivity to negative feedback was non-significant (*p* = 0.61), a common occurrence with relatively small samples. Mirroring the results reported above, childhood adversity was associated with left accumbofrontal tract integrity (*z* = −2.83, *p* = 0.005) and left Accumbofrontal tract integrity was associated with sensitivity to negative feedback (*z* = −3.03, *p* = 0.002). The indirect effect (a × b) was significant in the model containing the direct path from adversity and sensitivity to negative feedback (*B* = 0.01, SE = 0.006, *z* = 1.974, *p* = 0.048; 95% CI = 0.002–0.025). Indirect effect models for the right accumbofrontal tract were not significant (*B* = 0.009, SE = 0.005, *z* = 1.634, *p* = 0.102; 95% CI = 0.000–0.020, as shown in Fig. 3). These models were adjusted for age (in months), race (binary coded), general cognitive ability, and sex.

## Discussion

This study aimed to investigate the impact of ELA on the development of the accumbofrontal tract, a white matter pathway connecting the VS with the mPFC that has been implicated in adaptive reward learning. We found that adolescents who experienced higher levels of adversity during early childhood had lower accombofrontal tract integrity, as indexed by QA. The accombofrontal tract connects the VS and the mPFC—central hubs in the reward circuit [16]. Focusing in on this tract, we also found accombofrontal integrity predicted adolescents’ ability to use negative feedback in a reward learning task.

The present white matter connectivity findings are well situated with regard to published research on early adversity and neurobiology. Childhood adversity has been implicated in VS dysfunction, as well as a reduction in gray matter [20, 22, 63–65]. In addition, childhood adversity is associated with reduced mPFC volume and mPFC functional responsivity [18, 19, 54, 66]. These previously reported neurobiological differences may be a cause or a consequence of alterations in white matter connectivity. Lower white matter integrity may mean slower communication between the VS, mPFC, and other reward-processing brain areas, potentially leading to structural and functional alterations in these brain regions over time. Alternatively, initial structural or functional differences in the VS and mPFC could lead to alterations in white matter connectivity in the corticostriatal circuit. Future research should aim to increase understanding of these and connected neurobiological cascades related to adversity.

We focused on feedback sensitivity because a number of past studies have provided consistent evidence that children who experience severe adversity early in their lives evince deficits in elements of reward learning [8–10, 12, 67]. Here, we find associations between neurobiology and sensitivity to negative, but not positive, feedback. Such findings suggest that youth who experience ELA may be especially sensitive to forms of negative feedback (e.g., punishment), and that this feedback may do more harm than good in helping guide their future behavior. This type of increased sensitivity is often related to shifting or switching behavioral choices after a loss or a punishment [68, 69]. This behavioral tendency has been linked to depression [70], and may represent a link between childhood adversity and maladaptive responding to challenges and stress later in these individuals’ lives [53, 66]. Of note, while past investigations in adversity exposed samples have noted lower brain activity to positive feedback and stimuli [21, 54, 66], we did not find connections between positive feedback sensitivity, adversity, and white matter integrity. This may be due to the current paradigm’s inability to parse information about feedback valence (i.e., positive/negative) from uncertainty and risk (e.g., likelihood of winning versus not) [71]. Future work will need to be attentive to these distinctions and could be well-poised to test emerging theories about adversity influencing the parsing and processing of uncertainty in decision-making [71, 72].

Our work is not without limitations. First, our study design could be leading to underestimations of the full effects of adversity on children’s development. Participants were a community recruited, rather than a high risk, sample. Therefore, a significant proportion of these youth had limited exposure to adversity. Surveying more extreme groups (i.e., scores of 1 vs. 10 on the YSLI) in future research might reveal the full magnitude of adversity’s impact on neurobiology and reward-learning. Second, the project had a modest sample size, limiting aspects of the work and was therefore underpowered to fully test causal relations between childhood adversity, brain connectivity, feedback sensitivity, and behavioral differences in youth (e.g., choice behavior on our experimental task). Similarly, our statistical mediation models focused on a small number of variables that were significant in linear regression models. This could be increasing the probability of finding evidence of statistical mediation in our study. Third, the direct effect of ELA on reward sensitivity was not significant, but we investigated indirect effects of this association through accombofrontal integrity. The lack of a direct effect could be due to statistical power and the modest sample size of our study. This could also be due to the confluence of the multiple factors driving decision-making and reward-learning in our experimental paradigms (e.g., impulsivity, risk estimation, exploration/exploitation levels). All of these factors are related to reward sensitivity and may be influenced by adversity [73–77]. Finally, we isolated our white matter tracts of interest using an adult brain template from the Human Connectome Project. Past work suggest atlas-transformed brain morphology is relatively consistent across pediatric and adult samples [78]; however, youth with lower exposure to adversity could be fitting to average adult brain templates better than youth exposed to high life challenges.

Childhood adversity has been associated with the numerous aspects of brain development that have implications for behavior [79–81]. Here, we attempted to gather rich information about how children experienced adversity as a way to understand how and why the nervous system would respond over development (for review, see [1]). More specificity in understanding the mechanisms of development could provide more targeted prevention and intervention programs for children at risk for behavioral problems.

## FUNDING AND DISCLOSURES

Funding for this project was provided by grants from the National Institute of Mental Health (MH61285 to SDP, and MH84051 and MH43454 to RJD), the National Institute of Child Health and Human Development (U54 HD090256 to SDP), and the National Institute of Drug Abuse (DA028087 to JLH). RJD is the founder, president, and serves on the board of directors for the non-profit organization, Healthy Minds Innovations, Inc. The remaining authors have nothing to disclose.

## Supplementary information

Supplemental Materials
