# Supplementary material for: Accumbofrontal tract integrity is related to early life adversity and feedback learning
Source: Neuropsychopharmacology. 2021 Sep 24;46(13):2288–94. doi: 10.1038/s41386-021-01129-9 (PMC8581005; doi:10.1038/s41386-021-01129-9)
Supplement: Supplementary file 1 — Supplemental Materials [file 41386_2021_1129_MOESM1_ESM.docx]

**Supplemental Materials for “*Accumbofrontal Tract Integrity is Related to Early Life Adversity and Feedback Learning*”**

*Additional Information Related to the Mathematical Modeling of Learning*

To assess subcomponents of reward learning, a reinforcement learning (RL) model was fit to each participant’s behavioral data [S1]. This approach is commonly employed in decision-making research with adults [S2,S3]. RL models use the prediction error (δ) to update the decision weights (w) associated with each stimulus (in this case A, B, C, or D). Thus, whenever feedback is better than expected, the model will generate a positive prediction error, which is used to ‘‘increase’’ the decision weight of the chosen stimulus (e.g., stimulus A). However, when feedback is worse than expected, the model will generate a negative prediction error, which is used to ‘‘decrease’’ the decision weight of the chosen stimulus (e.g., stimulus B). The impact of the prediction error is scaled by a feedback sensitivity parameter (α), which we calculated for positive feedback (α _pos_) and negative feedback (α _neg_). Past investigations have operationalized these α-parameters as ‘learning rates;’ however, we construe these data as reflecting how much subsequent behavior is updated based on feedback. Thus, we refer to this parameter using the term feedback sensitivity in presenting our results.

To model trial-by-trial choices, we used a softmax function to compute the probability (P) of choosing a highly rewarded stimulus (A or C) on trial t. The difference in the decision weights in each trial (w_t_) associated with each stimulus was then passed through a sigmoid function. Sigmoidal and softmax normalization reduce the influence of extreme values (or unexpected choice) in the data without removing them from the dataset. For example, when stimulus pair AB is presented, the probability of choosing A is determined by:

$${P\left( A \right)}_{t}= \frac{1}{1+e^{-\beta\cdot(w \left( A \right)_{t}-w {(B)}_{t)}}}$$

where the inverse temperature function (β) here accounts for the randomness, or stochasticity, of an individual’s choices. Such models are drawn from the study of rates of chemical kinetics, hence the use of temperature in analytic models. In this field, actions with nearly the same probability have ‘high temperatures,’ whereas actions where the probabilities are more affected by environmental conditions (in this case expected rewards) have ‘lower temperatures.’ After each decision, the prediction error (δ) is calculated as the difference between the outcome received (r = 1 for positive feedback and 0 for negative feedback) and the decision weight (w_t_) for the chosen stimulus:

$$\delta_{t}=r_{t}-w(chosen\_stimulus)$$

Subsequently, the decision weights are updated according to:

$$w_{t}=w_{t}+ \lambda\times{\alpha(outcome)}_{t}\times\delta_{t}$$

where $\lambda$ is 1 for the chosen and 0 for the unchosen stimulus, $\alpha$(outcome) is a set of feedback sensitivities for positive (α_pos_) and negative feedback (α_neg_), which scale the effect of the prediction error on the future decision weights and thus subsequent decisions. For example, a high sensitivity for positive feedback but a low sensitivity for negative feedback indicates that positive feedback has a high impact on future behavior, whereas negative feedback will hardly change future behavior. To understand each participant’s expectation of reward, we employed two approaches to behavioral modeling. In line with past work, we first set the decision weights (w) for each stimulus to the same value at the beginning of the experiment. This would indicate that all participants had the same expectation of rewards for each stimulus. In addition, we also modeled this as a free parameter. By explicitly modeling this, it would allow us to capture individual differences in initial expectations of rewards. The 2 feedback sensitivity parameters and the initial decision weight were individually estimated by fitting the model predictions to participants’ actual decisions.

To compare different model factors including the initial expectations of rewards and sensitivity to different types of feedback, we used a robust combination of grid-search (100 different starting points) and the multivariate constrained minimization function (fmincon) of the optimization toolbox implemented in MATLAB 2012b (MathWorks, Inc., Natick, Massachusetts, USA). These two algorithms divided different model factors into a grid and evaluated possible combinations. Each point on the grid served as a starting position for the minimization function. This was then used to find the parameters that maximize the model fit of an individual’s decision, as measured by the log likelihood for an individual. The grid point that produced the maximum fit over all starting positions was selected for the final solution.

*Exploratory Analyses Related to “Stress-Inoculation” Effects*

While the negative impacts of early life adversity are well-known and well-documented, a burgeoning body of research suggest that adversity, in modest amounts, can lead to the subsequent development of resilience [S4]. For example, non-human primates exposed to challenging, but not overwhelming, stressful events early in life, show less anxiety and lower hypothalamic–pituitary–adrenal axis responsivity later in life [S5]. Similarly, moderate, but not extreme, levels of early adversity are related to larger volumes and greater activity in the prefrontal cortices of non-human primates [S6,S7].

Motivated by these ideas, we constructed exploratory regression models to examine if stress exposure might be showing non-linear effects. White matter integrity for Accombofrontal tracts was entered as our dependent variable (left and right entered separately in 2 different models), while mean-centered and squared YLSI interview scores were entered as our independent variable. This transformed variable would represent a more curvilinear effect in line with stress-inoculation models. Age, sex, and race were also entered into our models as covariates of no interest. Interestingly, mean-centered and squared YLSI interview scores were related to Accumbofrontal tract integrity in both the left (*β=- -0.361, p= 0.0025*) and right (*β= -0.439, p= 0.0001*) hemispheres. We then used the ‘*psych’* R library to test for potential differences between two models with highly correlated variables (original YLSI interview scores and mean-centered and squared YLSI interview score, *r= 0.598*). This comparison indicated that neurobiological associations with the original YLSI interview scores and mean-centered and squared YLSI interview scores were not significantly different from one another (left Accumbofrontal tract *t=1.18, p<0.24*; right Accumbofrontal tract *t=0.71, p<0.48*).

**Supplemental References**

S1. Sutton RS, Barto AG, others. Introduction to reinforcement learning. vol. 135. MIT press Cambridge; 1998.

S2. Cohen MX, Ranganath C. Reinforcement learning signals predict future decisions. J Neurosci. 2007;27:371–378.

S3. Gläscher JP, O’Doherty JP. Model-based approaches to neuroimaging: Combining reinforcement learning theory with fMRI data. Wiley Interdiscip Rev Cogn Sci. 2010;1:501–510.

S4. Lyons DM, Parker KJ. Stress inoculation-induced indications of resilience in monkeys. J Trauma Stress. 2007;20:423–433.

S5. Parker KJ, Buckmaster CL, Schatzberg AF, Lyons DM. Prospective Investigation of Stress Inoculation in Young Monkeys. Arch Gen Psychiatry. 2004;61:933–941.

S6. Lyons DM, Afarian H, Schatzberg AF, Sawyer-Glover A, Moseley ME. Experience-dependent asymmetric variation in primate prefrontal morphology. Behav Brain Res. 2002;136:51–59.

S7. Parker KJ, Buckmaster CL, Justus KR, Schatzberg AF, Lyons DM. Mild early life stress enhances prefrontal-dependent response inhibition in monkeys. Biol Psychiatry. 2005;57:848–855.
